# Integrative analysis of non-targeted metabolome and transcriptome reveals the mechanism of volatile formation in pepper fruit

**DOI:** 10.3389/fgene.2023.1290492

**Published:** 2023-11-10

**Authors:** Yuhua Liu, Jiahao Zhou, Cheng Yi, Fengqingyang Chen, Yan Liu, Yi Liao, Zhuqing Zhang, Wei Liu, Junheng Lv

**Affiliations:** ^1^ College of Life Sciences, Hengyang Normal University, Hengyang, Hunan, China; ^2^ Hunan Key Laboratory for Conservation and Utilization of Biological Resources in the Nanyue Mountainous Region, Hengyang, Hunan, China; ^3^ Vegetable Institution of Hunan Academy of Agricultural Science, Changsha, Hunan, China; ^4^ College of Medical Technology, Hunan Polytechnic of Environment and Biology, Hengyang, Hunan, China; ^5^ Key Laboratory of Vegetable Biology of Yunnan Province, College of Landscape and Horticulture, Yunnan Agricultural University, Kunming, Yunnan, China

**Keywords:** pepper, volatile organic compounds, metabolome, transcriptome, transcription factors

## Abstract

**Introduction:** Aroma is a key inherent quality attributes of pepper fruit, yet the underlying mechanisms of aroma compound biosynthesis remain unclear.

**Methods:** In this study, the volatile profile of the QH (cultivated *Capsicum chinense*) and WH (cultivated *Capsicum annuum*) pepper varieties were putatively identified during fruit development using gas chromatography-mass spectrometry (GC-MS).

**Results and discussion:** The results identified 203 volatiles in pepper, and most of the esters, terpenes, aldehydes and alcohols were significantly down-regulated with fruit ripening. The comparison of volatile components between varieties revealed that aldehydes and alcohols were highly expressed in the WH fruit, while esters and terpenes with fruity or floral aroma were generally highly accumulated in the QH fruit, providing QH with a fruity odor. Transcriptome analysis demonstrated the close relationship between the synthesis of volatiles and the fatty acid and terpene metabolic pathways, and the high expression of the *ADH*, *AAT* and *TPS* genes was key in determining the accumulation of volatiles in pepper fruit. Furthermore, integrative metabolome and transcriptome analysis revealed that 208 differentially expressed genes were highly correlated with 114 volatiles, and the transcription factors of *bHLH*, *MYB*, *ARF* and *IAA* were identified as fundamental for the regulation of volatile synthesis in pepper fruit. Our results extend the understanding of the synthesis and accumulation of volatiles in pepper fruit.

## 1 Introduction

Pepper belongs to the Solanaceae family and is one of the most widely cultivated vegetables and spice crops in the world. According to FAO statistics, the cultivated area of pepper reached 1.99 million ha in 2017, with an annual output of 36.1 million tons, and the cultivated area continues to expand (Ge et al., 2020). Pepper fruit is rich in nutrients, including capsaicin, flavonoids, carotenoids and vitamin C, and is favored by consumers due to its bright color, spiciness and aroma (Taiti et al., 2019). Aroma is an important component of pepper fruit flavor, and the loss of aroma will directly affect the sensory quality of the fruit, thereby affecting its economic value. Therefore, it is of great significance to strengthen the identification of volatile aroma components and analyze the molecular mechanism of their biosynthesis for the breeding and improvement of aromatic pepper varieties.

Aroma, a key index for the evaluation of fruit and vegetable quality, originates largely from the volatile organic components (VOCs) released by fruits. To date, over 2000 VOCs have been identified from fruits or flowers, including esters, aldehydes, alcohols, terpenes, phenols, ketones, ethers and other categories (Yang et al., 2019). Moreover, based on the different sensory effects, VOCs can be grouped into fruity aroma, sweet aroma, floral aroma, green aroma, spicy aroma, woody aroma and other types (Dudareva et al., 2006). More than 100 types of VOCs have been identified in pepper, mainly compromising esters, terpenes, alcohols and aldehydes (Kollmannsberger et al., 2011). In a quantitative analysis of volatiles in 10 pepper varieties with different colors, Pino et al. (2007) revealed the content of total volatiles to range from 1.37 to 11.84 mg·kg^−1^ (dry weight), with orange and brown varieties exhibiting higher levels of esters and a stronger aroma than red varieties. Furthermore, *Capsicum chinense*, one of the main cultivated species of pepper, is richer in volatiles than other cultivated species. The high accumulation of esters such as hexyl 2-methylbutanoate, heptyl 2-methylpropanoate, hexyl 3-methylbutanoate, hexyl pentanoate, heptyl pentanoate in the fruit of *C. chinense* result in a strong fruity and floral aroma (Garruti et al., 2013; Taiti et al., 2019). The accumulation of volatiles in pepper fruit is not only specific to the variety, but also varies with the development stage of the same variety. In the early stage of fruit development, esters and alcohols are the main volatile organic components in pepper fruit, but their contents gradually decrease with the development of fruits, accompanied by the increase of alkenes, aldehydes and ketones (Forero et al., 2009).

Volatile organic components are secondary metabolites that are primarily produced by fatty acid and terpene metabolic pathways (Liu et al., 2021). In particular, aldehydes, alcohols and esters are synthesized from fatty acid metabolic pathways. First, glycerides are catalyzed by lipases to form the starting substrate, namely, free fatty acids. The oxidation, cracking and dehydrogenation reactions catalyzed by fatty acid desaturase (FAD), lipoxygenase (LOX), hydroperoxide lyase (HPL) and alcohol dehydrogenase (ADH) are then followed by the production of aldehydes and alcohols from unsaturated fatty acids. Alcohols can be further catalyzed by alcohol acyltransferase (AAT) to form branched-chain esters (Beekwilder et al., 2004). However, the synthesis of lactones is more complicated than that of branched-chain esters. First, fatty acids need to form four or five hydroxy fatty acid intermediates through β-oxidation, and subsequently form lactones under self-cyclization or AAT catalysis (Sánchez et al., 2013). Sarde et al. (2018) identified eight potential *LOX* genes, of which *CaLOX6* and *CaLOX7* were predicted to be involved in the biosynthesis of green leaf volatiles. Previous gene expression analysis experiments indicate that the high expression of *ADH*, *HPL*, and *LOX* induced by methyl jasmonate and hexanal promoted the production of α-farnesene and hexanoic acid in fruit (Padmanabhan et al., 2020; Magalhães et al., 2021). In addition to regulating the production of VOCs, *LOX* has also been reported to participate in abiotic stress, the defense of microbial pathogens and cell death reactions (Lim et al., 2015).

Terpenes are mainly hydrocarbons or oxygenated hydrocarbon derivatives with isoprene structure, and are synthesized through the mevalonic acid pathway (MVA) and methylerythritol phosphate pathway (MEP) (Ramya et al., 2020). Based on the number of isoprenes in the molecule, terpenes are divided into monoterpene (C10), sesquiterpene (C15), diterpene (C20), triterpene (C30), tetraterpene (C40) and polyterpene (n C5, n > 10). Isopentenyl diphosphate (IPP) and diphosphates dimethylallyl diphosphate (DMAPP) are the precursors of all terpenoids. They are produced through the MVA pathway in the cytoplasm and the MEP pathway in the plastid to form various terpenoids catalyzed by terpene synthase (TPS), such as D-limonene, nerolidol, linalool and β-ionone (Zhang et al., 2020). Moreover, some carotenoids such as α-carotene and β-carotene can also be cleaved into sesquiterpene ketones (α-ionone and β-ionone) by carotenoid cleavage dioxygenase (CCD) (Shi et al., 2021). Research on the synthesis of terpenoids in pepper is limited, and only three QTLs (*LG3*, *LG10.1* and *LG1*) have been associated with the synthesis of terpenoids in pepper fruit, of which *LG10.1* and *LG1* were identified to be involved in the accumulation of 15 monoterpenes including linalooloxide and p-menth-1-en-9-al (Eggink et al., 2013). It is worth noting that two sesquiterpene synthase (*sTPS1* and *sTPS2*) have been isolated from pepper fruit, yet their involvement in the synthesis of terpenoids requires clarification (Zhang et al., 2019).

As one of the key traits affecting pepper fruit quality, volatiles have been the subject of much research. However, the majority of studies focus on the identification of volatiles during postharvest processing, while research on the synthesis and regulation mechanism of volatiles during fruit development is limited. In recent years, with the continuous development of biological technology, multi-omics analysis has become one of the most effective methods for the analysis of plant physiological processes (Liu et al., 2020; Shi et al., 2021). In this study, two pepper varieties QH and WH, which are popular in Hunan, China, were identified as the experimental materials. Fruit of QH is wide and lantern-shaped, with strong aroma and high capsaicin, and is widely used for sauce, while fruit of WH is short and narrow, with poor aroma but moderately spicy, and is mainly used for fresh eating. We employed headspace solid-phase microextraction-gas chromatography-mass spectrometry (HS-SPME-GC-MS) to identify volatiles in two pepper varieties during fruit development, and subsequently adopted transcriptome sequencing technology to screen differentially expressed genes (DEGs) in these varieties. Finally, correlation analysis between transcripts and VOCs was performed to identify key genes involved in VOC biosynthesis.

## 2 Materials and methods

### 2.1 Plant material and sampling

**FIGURE 1:**
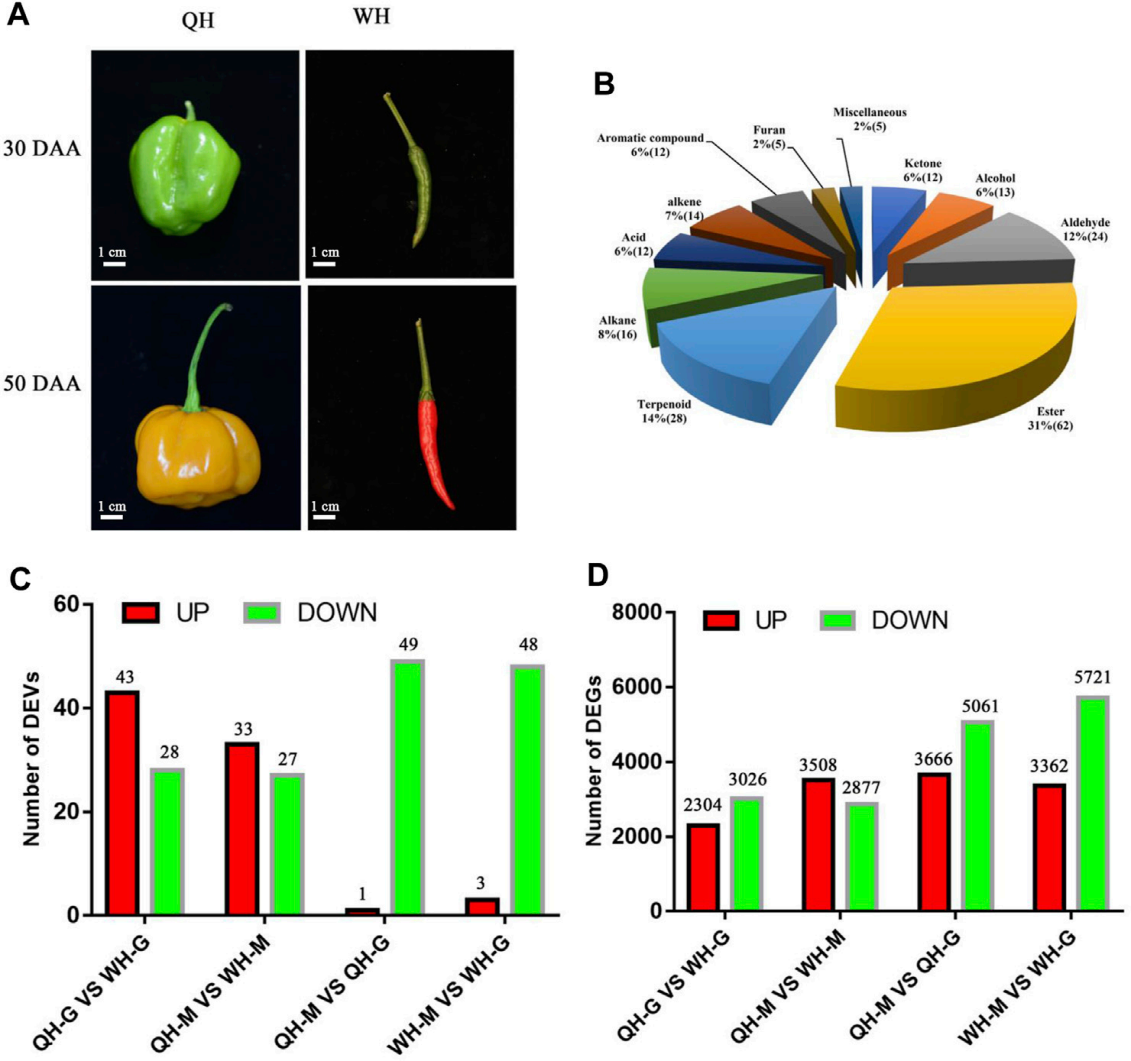
Fruit phenotype, types of volatiles and number of differentially expressed volatiles (DEVs) and differentially expressed genes (DEGs) between two pepper varieties. **(A)** Fruit phenotype of QH and WH varieties at 30 days after anthesis (DAA) and 50 DAA stages. **(B)** Volatile organic compounds detected in two pepper varieties and their classification. The number of DEVs **(C)** and DEGs **(D)** between two pepper varieties and two fruit developmental stages. QH-G, fruit samples of QH varieties at 30 DAA, QH-M, fruit samples of QH varieties at 50 DAA, WH-G, fruit samples of QH varieties at 30 DAA, WH-M, fruit samples of QH varieties at 50 DAA.

Two pepper varieties QH (*C. chinense* with a strong fruity aroma) and WH (*Capsicum annuum* without a fruity aroma) were selected as the research materials for this experiment (Figure 1A). After standardized seedling raising, the two pepper varieties were transplanted to the biological garden of Hengyang Normal University (Hengyang City, Hunan Province, China) for routine cultivation and management in March 2021. The peppers were sampled at 30 days after anthesis (30 DAA) and 50 days after anthesis (50 DAA). Each sample consisted of six fleshes from three plants (two fleshes per plant). The collected flesh was immediately frozen in liquid nitrogen for subsequent volatile detection (six biological replicates), transcriptome sequencing (three biological replicates) and qRT-PCR analysis.

### 2.2 Analysis of volatile organic compounds using GC–MS

Pepper fruit fleshes were weighed and ground to a powder in liquid nitrogen. Solid phase micro extraction (SPME) was used to extract the volatile compounds of fruit with six biological replicates for each sample. The fruit fleshes (500 ± 1 mg) was placed in a 20 mL headspace bottle, and 10 μL 2-octanol (100 mg/L stock in dH_2_O) was then added as an internal standard. The SPME cycle of the CTC rail system adopted the following analytical conditions: incubate temperature of 50°C, preheat time of 15 min, incubate time of 30 min, and desorption time of 4 min. Subsequently, samples were directly injected into the injection port of a GC-MS.

The volatile organic compounds of the pepper fruit were detected as described previously (Li et al., 2021a) with some modifications. The Agilent 7890 gas chromatograph system combined with 5975C mass spectrometer was employed to identify and quantify VOCs. Volatile analysis was performed using a HP-INNOWax capillary column (30 m × 250 μm × 0.25 μm, J&W Scientific, Folsom, CA, United States), with splitless mode used for the injection at 260°C. Helium (Purity ≥ 99.999%) was used as the carrier gas for the GC-MS operation, with a front inlet purge flow of 3 mL·min^−1^ and gas flow rate through the column of 1 mL·min^−1^. The initial oven temperature was maintained at 40°C for 1 min, raised to 250°C at a rate of 5°C·min^−1^, and maintained for 5 min at 250°C. The transfer line temperature, ion source temperature and quad temperatures were 260°C, 230°C, and 150°C, respectively. The energy of electron impact (EI) was −70 eV. The mass spectrometry data were acquired in scan mode with the m/z range of 20–400 and solvent delay of 0 min. Chroma TOF 4.3X (LECO Corporation) and the NIST and NCBI databases were used for the raw peak extraction, data baselines filtering, baseline calibration, peak alignment, deconvolution analysis, spectrum matching, peak identification and peak area integration. VOCs with MS matching scores greater than 700 were retained, and the final substance type was determined after manual identification and comparison. Furthermore, the identified VOCs were subjected to orthogonal partial least squares discriminant analysis (OPLS-DA), and VOCs with | Log2 (fold change) | ≥1 and *p*-value <0.05 were considered as differentially expressed volatiles (DEVs).

### 2.3 RNA-seq analysis

The total RNA was extracted from frozen fleshes. The integrity and total amount of RNA were accurately detected with an Agilent 2100 bioanalyzer (Agilent Technologies, CA, United States). The qualified RNA was used to construct a sequencing library. A total amount of 3 µg RNA per sample was used as input material for the RNA sample preparations. Sequencing libraries were generated using NEBNex NEBNext^®^ UltraTM RNA Library Prep Kit for Illumin Illumina^®^ (NEB, United States) following manufacturer’s recommendations and index codes were added to attribute sequences to each sample. Subsequently, the quality of the library was evaluated on the Agilent Bioanalyzer 2100 system (Agilent Technologies, CA, United States), and the qualified library was sequenced on the Illumina HiSeq 4000 platform. After sequencing, the adaptor and low-quality sequences were removed using Fastp software with default parameters, and then the obtained clean reads were aligned to the Zunla reference genome using Hisat2 (Chen et al., 2018). Following this, based on the gene length, the expected number of fragments per kilobase of transcript sequence per million base pairs sequenced (FPKM) value of each gene was calculated to determine the expression level of the corresponding gene. Finally, differential expression analysis between sample groups was performed with DESeq2 software (Love et al., 2014). Genes satisfying | log2Fold Change | ≥ 1 and False Discovery Rate (FDR) < 0.05 were defined as differentially expressed genes (DEGs), which were further analyzed using the Kyoto Encyclopedia of Genes and Genomes (KEGG) database.

### 2.4 Integrative analysis of the metabolome and transcriptome

The Pearson correlation coefficient was calculated for metabolome and transcriptome data integration. To reduce the false positive rate and accurately obtain the key genes regulating the synthesis of volatiles, differentially expressed genes with a FPKM value below 10 in all samples and | log2Fold Change | < 2 were filtered out. Previous studies have shown that the terpene metabolism pathway, fatty acid metabolism, plant hormone, cell signaling and transcription factors are key to determining the synthesis of plant volatiles (Ramya et al., 2020; Shi et al., 2021). Therefore, the differentially expressed genes (FPKM >10 and | log2Fold Change | > 2) in the aforementioned factors were used to calculate the Pearson correlation coefficient with volatiles with the “Wu Kong” platform (https://www.omicsolution.com/wkomics/main/). Correlations greater than 0.85 were selected and the relationships between the DEGs and VOCs were visualized using Cytoscape (version 3.7.1, Cytoscape Team).

### 2.5 qRT-PCR analysis

Total RNA was extracted from the fleshe of QH and WH using a MAGEN RNA Extraction Kit (Majorbio, China). The purified RNA was reverse transcribed into the first strand cDNA using the HiScript II 1st Strand cDNA Synthesis Kit (Vazyme, China). Synthesized cDNA was used as a template for qRT-PCR with a Real Master Mix (SYBR Green) kit (Vazyme, China). Nine genes related to volatile synthesis were selected for qRT-PCR analysis, with the β-actin gene used as the reference gene to correct gene expression. All primers used in this study are listed in Supplementary Table S1. qRT-PCR was conducted in a total volume of 20 µL containing 10 µL of mix, 0.4 µL of forward primer, 0.4 µL of reverse primer, 2 µL of cDNA, and 7.2 µL distilled water. Reactions were carried out on the LightCycler^®^ 96 Real-Time PCR detection system (Roche, Switzerland) with default parameters. Three replicates were performed for each sample. The relative expressions of target genes were calculated using the 2^−ΔΔCT^ method.

## 3 Results

### 3.1 Identification of volatiles in pepper fruit

The volatile organic compounds and relative changes in content of the QH and WH varieties were putatively identified during fruit development. A total of 203 VOCs were detected in the two pepper varieties, including 62 esters (31%), 28 terpenoids (14%), 24 aldehydes (12%), 16 alkanes (8%), 14 alkenes (7%), 13 alcohols (6%), 12 ketones (6%), 12 acids (6%), 12 aromatic compounds (6%), 5 furans (2%) and 5 miscellaneous (2%) (Figure 1B, Supplementary Figure S1; Supplementary Table S2). The hierarchal clustering of the VOC profile showed that the 203 volatiles could generally be classified into three categories (Supplementary Figure S2). The first cluster contained 76 VOCs, such as esters, terpenes and alkenes, which are mainly accumulated in the QH variety. The second cluster was composed of 14 VOCs including terpenoids, alkenes and aromatic compounds, which were highly expressed in the 50 DAA stage of the WH variety, showing significant variety and time specificity. The 113 VOCs (including aldehydes, alcohols and terpenes) in the third cluster exhibited a higher accumulation in the 30 DAA stage of the WH variety (Supplementary Figure S2). Thus, the differences in the contents of esters, terpenes, aldehydes and alcohols between the two pepper cultivars were responsible for the differences in fruit odor.

### 3.2 Comparison of differential VOCs between varieties and stages

**FIGURE 2:**
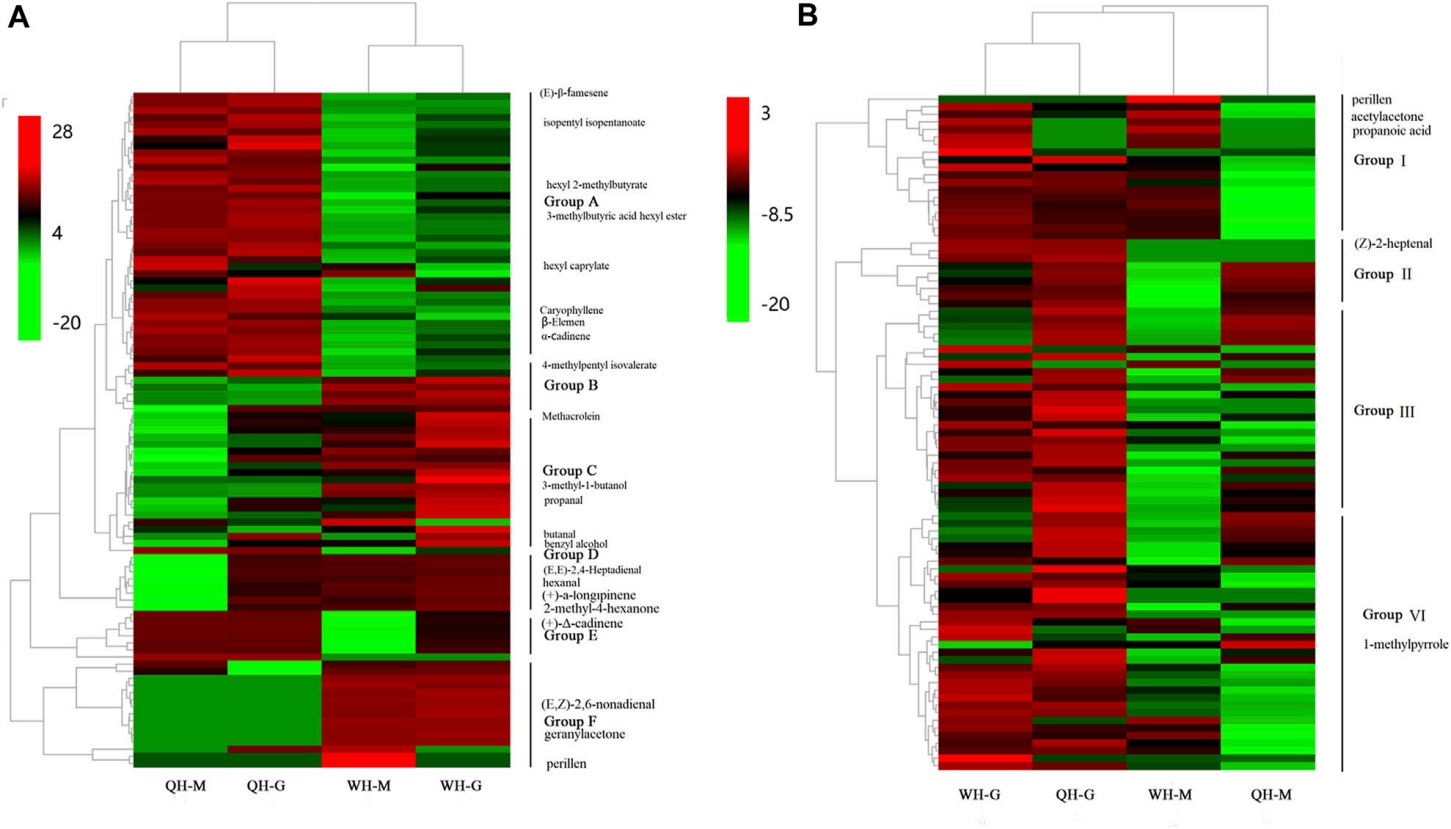
Heat map of differentially expressed volatiles. **(A)** Heat map of differentially expressed volatiles between varieties. **(B)** Heat map of differentially expressed volatiles between developmental stages. QH-G, fruit samples of QH varieties at 30 DAA, QH-M, fruit samples of QH varieties at 50 DAA, WH-G, fruit samples of QH varieties at 30 DAA, WH-M, fruit samples of QH varieties at 50 DAA.

Pairwise comparisons were performed between QH and WH at each developmental stage. In the 30 DAA and 50 DAA stages, 71 (43 upregulated, 28 downregulated) and 60 (33 upregulated, 27 downregulated) DEVs were detected between the QH and WH varieties, respectively (Figure 1C and Supplementary Table S3). After removing the redundancy, a total of 91 DEVs were detected between the two varieties, including esters (34), aldehydes (16) and terpenes (11) (Supplementary Table S4). To visually compare the differences of VOCs between the two varieties, hierarchical clustering was performed on the aforementioned 91 DEVs. As shown in Figure 2, a total of 91 DEVs were clustered into six groups (A, B, C, D, E and F) in the heatmap dendrogram (Supplementary Table S4). Group A consisted of 37 DEVs, including esters (21), acids (5) and terpenes (4). The terpenes and esters in group A, such as hexyl 2-methylbutyrate, 3-methylbutyric acid hexyl ester (hexyl 3-methylbutanoate), isopentyl isopentanoate, isobutyl isovalerate, oxacyclotetradecan-2-one, hexyl caprylate, (E)-β-famesene, caryophyllene, β-elemen and α-cadinene, exhibited a higher accumulation in the QH variety (Figure 2A and Supplementary Table S4). Consistent with group A, the esters were the main components in group B, with the exception of 4-methylpentyl isovalerate and cis-3-hexenyl 2-methylbutanoate, which were highly accumulated in the WH variety. Aldehydes and alcohols were the dominant components of group C, with nine aldehydes (methacrolein, 2-propenal, isovaleraldehyde, isobutyraldehyde, 3-methyl-2-butenal, trans-2-pentenal, hexadecanal, propanal and butanal) and two alcohols (3-methyl-1-butanol and benzyl alcohol) exhibiting a higher content in the WH fruit (Figure 2A and Supplementary Table S4). Group D, consisting of seven compounds (E-2-hexadecacen-1-ol, benzaldehyde, 7-tetradecene, (E, E)-2,4-heptadienal, hexanal, (+)-a-longipinene and 2-methyl-4-hexanone), presented a high content in the QH and WH fruit at 30 DAA, while at 50 DAA they were not detected in the QH (Figure 2A and Supplementary Table S4). Group E consisted of 6-methylheptyl 3-methylbutanoate, octyl-3-methylbutanoate, 6-methylhept-4-en-1-yl 3-methylbutanoate, (+)-Δ-cadinene, α-calacorene and cis-9-Hexadecenal, which were abundant in the QH and WH fruit at 30 DAA, but not detected in the WH fruit at 50 DAA (Figure 2A and Supplementary Table S4). Note that 15 VOCs in group F, such as 5-ethylcyclopent-1-enecarboxaldehyde, (E, Z)-2,6-nonadienal, perillen, pentanoic acid, acetylacetone, geranylacetone and pentanoic acid, were also highly expressed in the WH variety, 12 of which were not detected in the QH variety (Figure 2A and Supplementary Table S4).

During fruit development, 51 DEVs (48 downregulated and 3 upregulated) and 50 DEVs (49 downregulated and 1 upregulated) were detected in the WH and QH varieties between 30 DAA and 50 DAA, respectively (Figure 1C and Supplementary Table S3). After removing redundancy, a total of 88 DEVs were detected in the two pepper varieties between the two fruit development stages, including esters (32), aldehydes (15), alkenes (9), terpenes (8) (Supplementary Table S4). These DEVs were downregulated during fruit development, with the exception of 1-methylpyrrole, propanoic acid, perillen and acetylacetone (Supplementary Table S4). The hierarchical clustering of the DEVs showed that 88 volatiles could be divided into four groups (groups I, II, III, and VI) (Figure 2B and Supplementary Table S4). Group I contained 19 volatiles, of which aldehydes, alcohols and ketones were the main components. During fruit development, the volatiles of group I were significantly downregulated in the QH variety, while 16 of which were not detected in the QH fruit at 50 DAA (Figure 2B and Supplementary Table S4). In contrast to group I, the content of nine volatiles in group II decreased significantly in the WH fruit during fruit development, with (Z)-2-heptenal, (R)-β-himachalene, 6-methylheptyl 3-methylbutanoate, 2-methyl-1-propyl butyrate, resorcinol monobenzoate, (+)-a-longipinene and α-calacorene undetected in the WH fruit at 50 DAA (Figure 2B and Supplementary Table S4). The group C (26 members) and group D (34 members) volatiles exhibited a similar accumulation trend during the fruit development of the QH and WH varieties (Figure 2B and Supplementary Table S4). In the early stage of fruit development, the volatiles of groups C and D were highly abundant in the fruit of QH and WH, yet their content decreased slightly with fruit ripening.

### 3.3 Transcriptome analysis of the developing pepper fruit

The fruit samples of the two varieties were subjected to RNA-Seq analysis, with each sample run in triplicate. A total of 12 libraries were constructed, yielding 88.84 GB of clean reads (Supplementary Table S5). These clean reads were mapped to the reference genome with match ratios in the range of 88.94%–94.96%, and 26,366 genes predicted from the genome were found to be expressed in at least one sample (with FPKM >0) (Supplementary Tables S5, S6).

**FIGURE 3:**
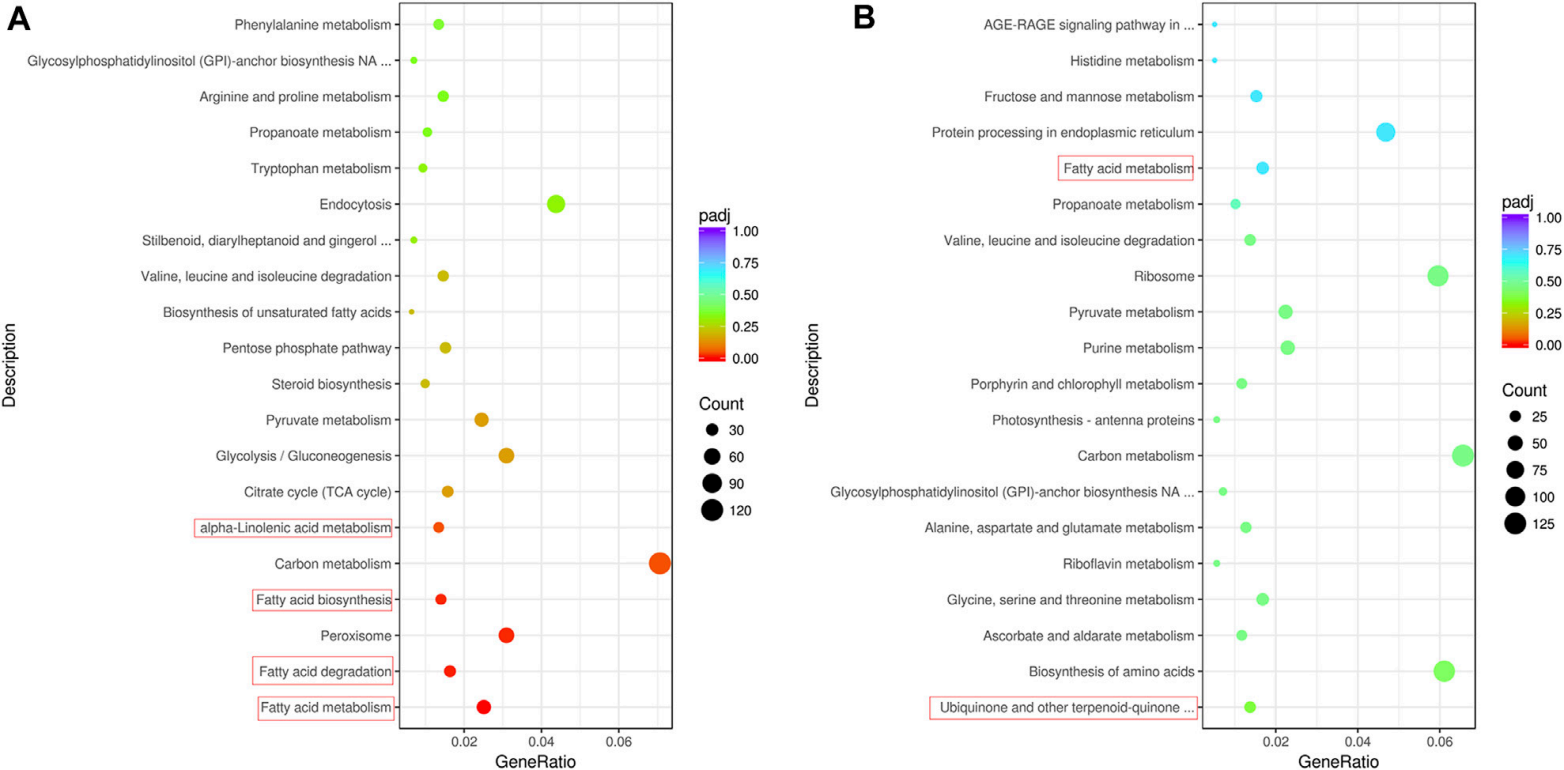
Enrichment pathway of differentially expressed genes. **(A)** Top 20 enriched pathways of differentially expressed genes between QH and WH varieties at 30 days after anthesis. **(B)** Top 20 enriched pathways of differentially expressed genes between QH and WH varieties at 50 days after anthesis.

To further screen the genes related to the formation of fruit volatiles, the differential expression analysis of genes was performed. At 30 DAA and 50 DAA, 5330 (2304 upregulated, 3026 downregulated) and 6385 (3508 upregulated, 2877 downregulated) DEGs were detected between QH and WH, respectively (Figure 1D and Supplementary Table S7). After removing redundancy, a total of 8,814 DEGs (differentially expressed at least one developmental stage) were identified between the two varieties, 2901 of which were differentially expressed at 30 DAA and 50 DAA (Supplementary Figure S3). KEGG enrichment analysis identified “fatty acid metabolism”, “fatty acid degradation”, “fatty acid biosynthesis”, “peroxisome”, “carbon metabolism” and “a-linolenic acid metabolism” as the six most significant pathways enriched with the DEGs at 30 DAA (Figure 3A). In 50 DAA, the DEGs were concentrated in “ubiquinone and other terpenoid-quinone biosynthesis”, “biosynthesis of amino acids”, “carbon metabolism”, “fatty acid metabolism”, and “pyruvate metabolism” (Figure 3B). Thus, the significant enrichment of DEGs in the fatty acid and terpene metabolic pathways may be responsible for the large differences in the fatty acid and terpene contents between QH and WH.

During fruit development, 9083 (3362 upregulated, 5721 downregulated) and 8,727 (3666 upregulated, 5061 upregulated) DEGs were detected in the WH and QH varieties, respectively (Figure 1D and Supplementary Table S7). After removing redundancy, 12,655 genes were differentially expressed in at least one variety, and 5155 genes were differentially expressed in both QH and WH during fruit development (Supplementary Figure S3). To identify the significantly enriched pathways, the DEGs of QH and WH during fruit development were mapped to the KEGG database. The results showed that the DEGs of QH were mainly enriched in “fatty acid biosynthesis”, “biosynthesis of amino acids”, “carbon metabolism” and “pyruvate metabolism”, while those of WH were mainly enriched in “ascorbate and aldarate metabolism”, “photosynthesis”, “sulfur metabolism” and “carbon metabolism” (Supplementary Figure S4).

### 3.4 Expression changes in volatile compounds-related genes involved in the fatty acid metabolism pathway

**FIGURE 4:**
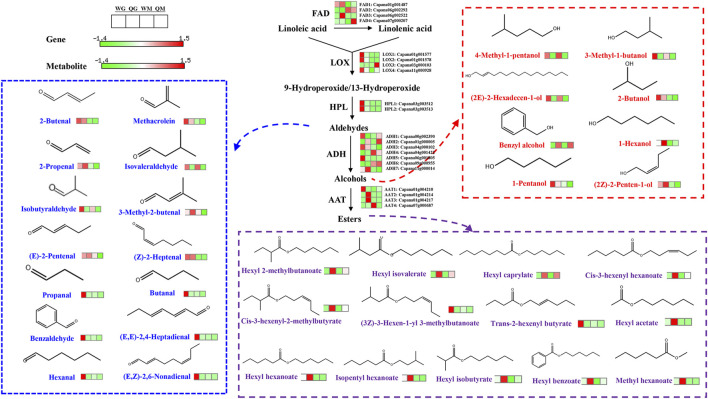
Volatile aldehyde, alcohol and ester biosynthesis in two pepper varieties fruit. Only esters, aldehydes and aldehydes with the most significant differential expression were displayed due to limited space. FAD, fatty acid desaturase, LOX, lipoxygenases, HPL, hydroperoxide lyase, ADH, alcohol dehydrogenase, AAT, alcohol acyltransferase.

Linoleic acid and linolenic acid are precursors for the synthesis of branched aliphatic alcohols, aldehydes, ketones and esters. In addition, linoleic acid can be converted into linolenic acid by fatty acid desaturase (FAD) (Liu et al., 2021). Four *FADs* were identified in QH and WH, and their expression patterns varied with the variety and developmental stage. *FAD1* was highly expressed at 30 DAA, while *FAD2* was highly expressed at 50 DAA. The remaining two *FAD* genes (*FAD3* and *FAD4*) exhibited significant specificity to the variety, and were only highly expressed at the 30 DAA and 50 DAA stages of QH, respectively (Figure 4 and Supplementary Table S8). Lipoxygenases (LOX) and hydroperoxide lyase (HPL) are key enzymes in the biosynthesis of aldehydes, and three *LOXs* (*LOX1*, *LOX2*, and *LOX4*) and two *HPLs* (*HPL1* and *HPL2*) were highly expressed in the WH fruit at the 30 DAA stage. This could be directly related to the high accumulation of aldehydes (e.g., hexanal, propanal, butanal, benzaldehyde, (E, E) −2,4-heptadienal and (E, Z) −2,6-nonadienal) in the WH fruit (Figure 4 and Supplementary Table S8). The aldehydes can be further converted into alcohols by alcohol dehydrogenase (ADH) (Wang et al., 2022a). In the WH fruit, the expressions of *ADH1*, *ADH3, ADH4,* and *ADH5* exceeded those in QH, while the reverse was true for *ADH2* and *ADH7* (Figure 4 and Supplementary Table S8). This may be the reason for the differential accumulation of 3-methyl-1-butanol, (2E) -2-hexadecen-1-ol, 1-pentanol, 4-methyl-1-pentanol, benzyl alcohol and 1-hexanol in the two varieties. Alcohol acyltransferase (AAT) are the rate limiting enzymes for ester biosynthesis. Here we identified four *AAT* genes, which were differentially expressed between varieties and developmental stages. In the early stage of fruit development, *AAT1*, *AAT2,* and *AAT3* were highly expressed in the QH and WH varieties, ensuring the accumulation of esters in the WH and QH fruit at 30 DAA, such as hexyl 2-methyl butyrate, isoamyl hexanoate, hexyl acetate and trans-2-hexenyl butyrate (Figure 4 and Supplementary Table S8). However, note that the *AAT4* gene exhibited the highest transcriptional abundance in the WH fruit at 50 DAA, while the ester content was extremely low in this variety at 50 DAA.

### 3.5 Expression changes in volatile compounds-related genes involved in the terpene metabolism pathway

**FIGURE 5:**
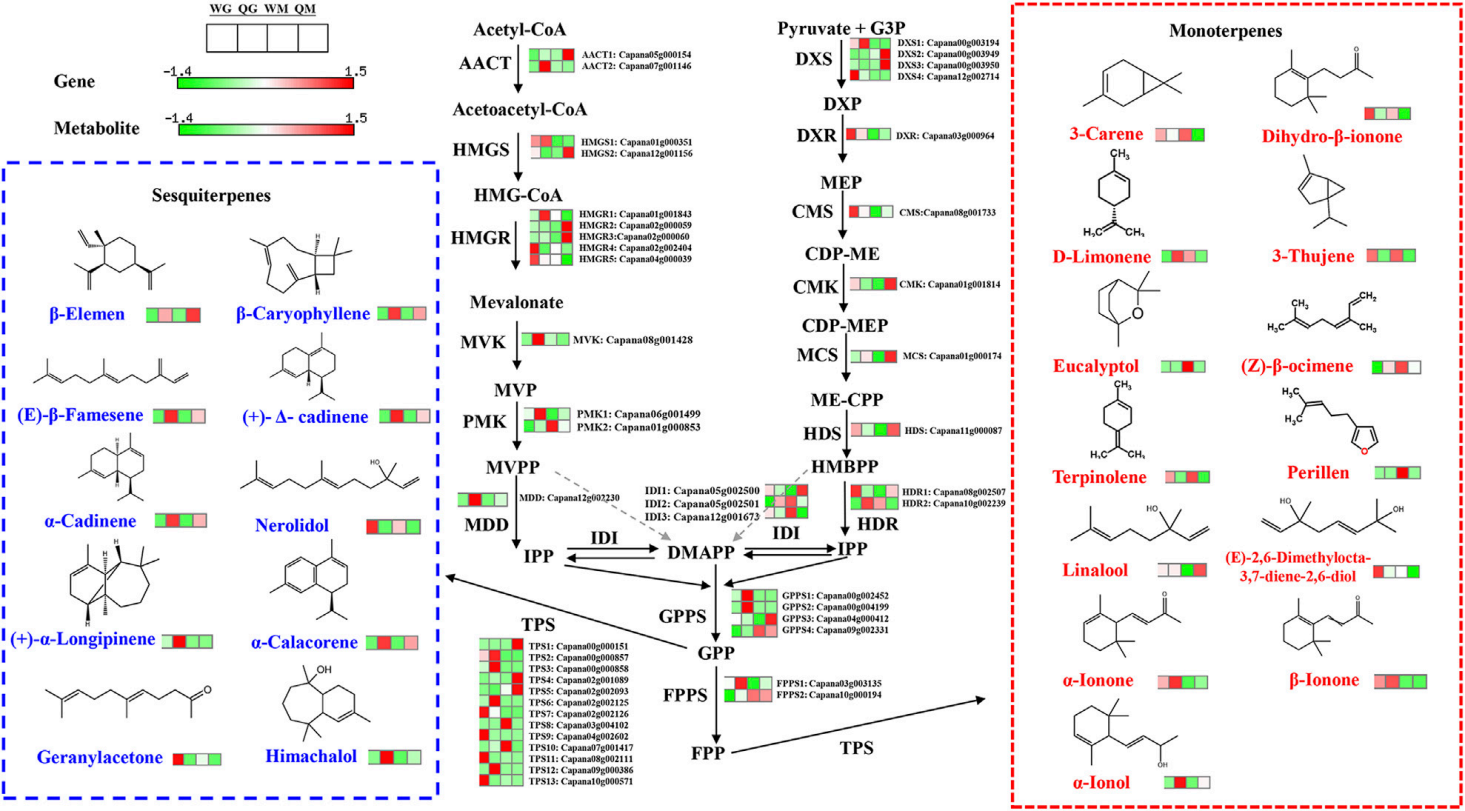
Volatile terpenoid biosynthesis in two pepper varieties fruit. Terpenoid volatile organic compounds are synthesized by the cytosolic mevalonic acid (MVA) and plastidial methylerythritol phosphate (MEP) pathways. Differentially expressed genes and volatiles were integrated into pathway-based maps. Dashed arrows indicate unverified steps in the pathway. HMG-CoA, 3-hydroxy-3-methylglutaryl coenzyme A, MVP, 5-phosphomevalonate, MVPP, 5-diphosphomevalonate, IPP, isopentenyl diphosphate, DMAPP, dimethylallyl diphosphate, DXP, 1-Deoxy-D-xylulose 5-phosphate, CDP-ME, 4-(cytidine 5′-diphospho)-2-C-methyl-D-erythritol, CDP-MEP, CDP ME 2-phosphate, ME-CPP, 2-C-methyl-D-erythritol 2,4-cyclodiphosphate, HBMPP, hydroxymethyl-butenyl 4-diphosphate, FPP, farnesyl diphosphate, GPP, geranyl diphosphate, AACT, acetoacetyl-CoA thiolase, HMGS, 3-hydroxy-3-methylglutaryl-CoA synthase, HMGR, HMG-CoA reductase, MVK, mevalonate kinase, PMK, phosphomevalonate kinase, MDD: Mevalonate 5-diphosphate decarboxylase, IDI, isopentenyl diphosphate isomerase, DXS, 1-deoxy-d-xylulose 5-phosphate synthase, DXR, 1-deoxy-d-xylulose 5-phosphate reductoisomerase, CMS, CDP-ME synthase, CMK, CDP-ME kinase, MCS, ME-CPP synthase, HDS, hydroxymethyl-butenyl 4-diphosphate synthase, FPPS, farnesyl pyrophosphate synthase, GPPS, geranyl pyrophosphate synthase, TPS, terpene synthase.

Terpenes are a large group of plant aromatic components and are mainly synthesized through the mevalonic acid (MVA) pathway in the cytoplasm and the methylerythritol phosphate (MEP) pathway in the plastids (Shi et al., 2021). During fruit development, the transcriptional abundance of 3-hydroxy-3-methylglutaryl-CoA synthase 1(*HMGS1*), 3-hydroxy-3-methylglutaryl-CoA reductase 5 (*HMGR5*), phosphomevalonate kinase 1 (*PMK1*) and mevalonate 5-diphosphate decarboxylase (*MDD*) decreased by varying degrees in the two pepper varieties, and the expression level of *HMGS1* in WH and QH at 30 DAA was 5.9 and 12.3 times higher than that at 50 DAA, respectively (Figure 5 and Supplementary Table S8). The expression levels of genes in the MVA pathway are not only different during fruit development, but also vary greatly among varieties. The transcription levels of acetoacetyl-CoA thiolase 2 (*AACT2*), *HMGR1* and *PMK1* in QH were significantly higher than those in WH at the 30 DAA stage, while the transcription levels of *HMGR1* and *HMGR5* in QH were lower than those in WH at 50 DAA (Figure 5 and Supplementary Table S8). In addition, four upstream structural genes (1-deoxy-d-xylulose 5-phosphate synthase 1(*DXS1*), *DXS4*, 1-deoxy-d-xylulose 5-phosphate reductoisomerase (*DXR*) and CDP-ME synthase (*CMS*) in the MEP were downregulated during fruit development, thus reducing the flux into the terpene synthesis pathway with the decrease in terpenes content in the pepper fruit at 50 DAA. Geranyl pyrophosphate synthase (GPPS) is a key downstream gene in the terpene pathway that is able to convert isopentenyl pyrophosphate (IPP) or dimethyl allyl pyrophosphate (DMAPP) into geranylgeranyl pyrophosphate (GPP), the precursor of terpene synthesis (Zhang et al., 2020). We identified four *GPPS* genes (*GPPS1*-*GPPS4*), of which *GPPS1*, *GPPS2* and *GPPS3* exhibited a high transcriptional abundance in QH, while *GPPS4* was highly expressed in WH (Figure 5 and Supplementary Table S8).

Terpene synthase (TPS) is a large family that determines the synthesis of different terpenoids in plants. In this study, 13 genes were predicted to encode TPS, and their transcription showed significant variety specificity. Seven *TPS* (*TPS1*-*TPS6* and *TPS12*) had the highest transcription abundance in QH, while the remaining six *TPS* (*TPS7*-*TPS11* and *TPS13*) were highly expressed in WH (Figure 5 and Supplementary Table S8). Note that the accumulation of terpenes also exhibited significant variety specificity. Monoterpenes, such as 3-carene, 3-thujene, eucalyptol, terpinolene and perillen, were mainly accumulated in WH, while sesquiterpenes, such as β-elemen, β-caryophyllene, (E)-β-famesene, (+)-Δ-cadinene, α-cadinene, α-calacoren and (+)-α-longipinene, were highly expressed in QH (Figure 5 and Supplementary Table S8). Therefore, the consistency of the *TPS* expression level and terpenoid content implies that *TPS1*-*TPS6* and *TPS12* may be mainly involved in the synthesis of sesquiterpenes in pepper fruit, while the remaining six *TPS* genes (*TPS7*-*TPS11* and *TPS13*) may be primarily responsible for the synthesis of monoterpenes.

### 3.6 Correlation analysis between transcripts and VOCs identifies key genes involved in VOC biosynthesis

**FIGURE 6:**
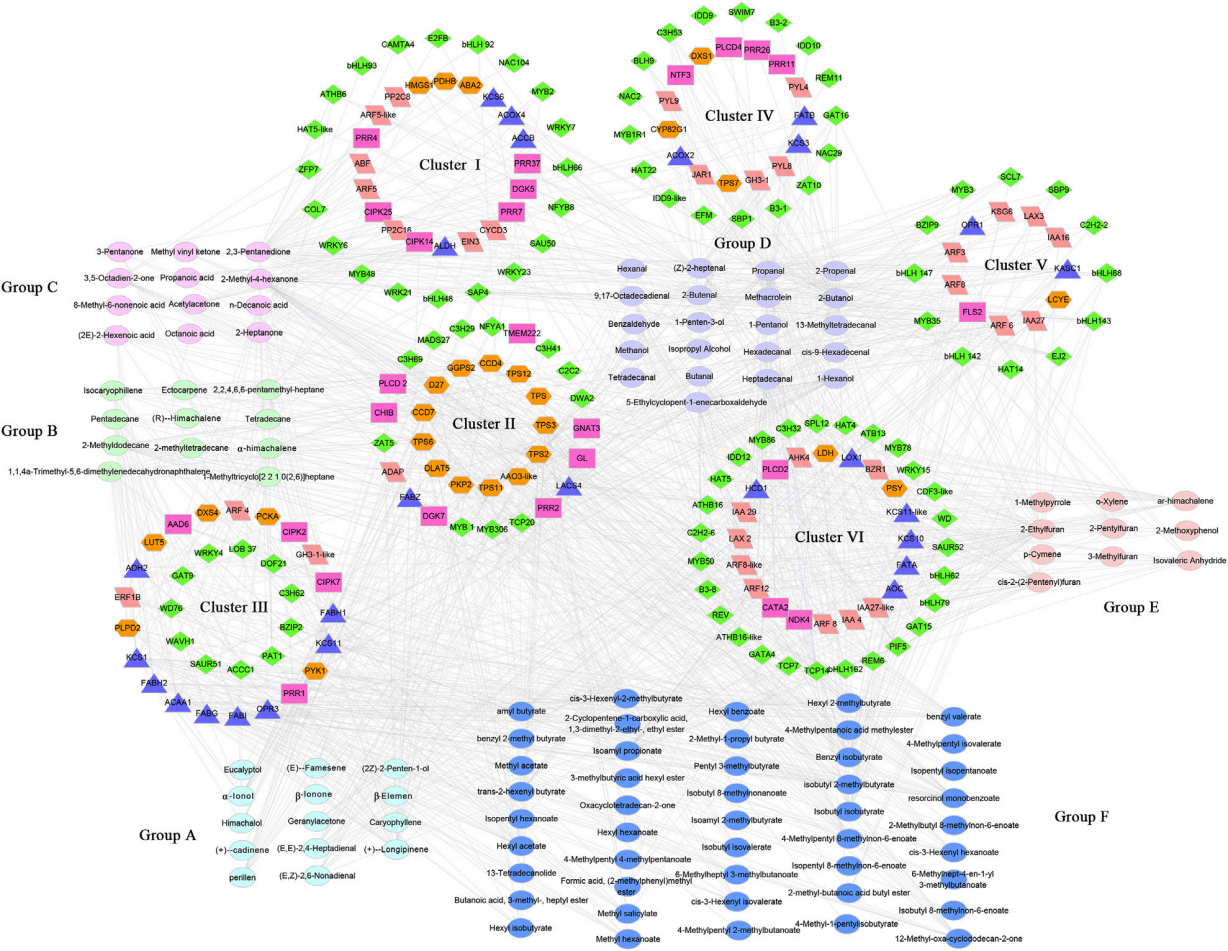
The correlation networks of volatiles and differentially expressed genes (FPKM >10 and | log2Fold Change | > 2) in terpenes metabolism, fatty acids metabolism, hormones metabolism, cell signaling and transcription factors. Triangle, fatty acid metabolism genes (blue), Hexagon, Terpene metabolism genes (orange), Rectangle, cell signaling genes (purplish red), Rhombus, transcription factors (green), Parallelogram, hormones metabolism genes (pink).

To understand the regulation mechanism of volatile biosynthesis, we performed correlation tests between the quantitative changes of metabolites and transcripts in the QH and WH pepper varieties. A total of 203 VOCs and 356 DEGs (43 for terpene metabolism, 43 for fatty acid metabolism, 50 for hormone metabolism, 44 for cell signaling, 176 for transcription factors, Supplementary Table S9) were subjected to Pearson correlation analysis. A total of 114 VOCs were strongly correlated with 208 DEGs (PCC > 0.85). These 114 volatiles were grouped into six groups (groups A-F) and the 208 genes were divided into six clusters (clusters I-VI) (Figure 6 and Supplementary Table S10).

Group A, consisting of 14 terpenoids, exhibited a strong correlation with the cluster II gene. In cluster II, 13 terpene metabolism-related genes (*GGPS2, D27, CCD7, CCD4, DLAT5, PKP2, AAO3-like, TPS, TPS2, TPS3, TPS6,* and *TPS11*) had a high correlation with the VOCs in group A, contributing 26.3% to the correlation in group C (Figure 6). *CCD* is a structural gene in the carotenoid metabolic pathway that can cleave carotenoids into low-carbon terpenes (Shi et al., 2021). In cluster II, *CCD4* and *CCD7* were strongly correlated with (+)-α-longipinene and α-ionol (PCC >0.9), indicating the possible key roles of these genes in the production of (+)-α-longipinene and α-ionol (Figure 6). Moreover, six *TPS* genes (*TPS1, TPS2, TPS3, TPS6, TPS11,* and *TPS12*) were identified in cluster II, but their associated volatile components were different. *TPS1, TPS2, TPS3, TPS6,* and *TPS12* mainly interacted with α-ionol, β-ionone and (+)-α-longipinene, while *TPS11* was associated with geranylacetone, (E, E) −2,4-heptadienal and (E, Z) −2,6-nonadienal (Figure 6), furthering proving that these six *TPS* genes have potential functions in regulating terpene synthesis. In addition to terpene metabolism genes, 27 transcription factors, such as *bHLH142, bHLH147, bZIP9, MYB1, MYB2, MYB306, MYB35,* and *WRKY4*, were also identified to have a strong correlation with geranylacetone, (E, Z)-2,6-nonadienal, perillen, (+)-α-longipinene and (E, E)-2,4-heptadienal (Figure 6).

Group B, consisting of six alkanes and five alkenes, was mainly related to cluster III and cluster VI genes (Figure 6). Fatty acid metabolism-related genes were significantly enriched in cluster III, and nine genes, including *ADH2,* acetyl-CoA acyltransferase 1 (*ACAA1*), β-ketoacyl-CoA synthase 1 (*KCS1*) and *KCS11*, were highly correlated with the volatiles in group B, contributing 26.6% correlation of group B. In cluster VI, transcription and hormone response factors including *bHLH162, ARF8, ARF12, ARF8-like, TCP14, GATA4, C2H2-6,* and *REV* were associated with group B volatiles, and were only correlated with 2-methyldodecane (Figure 6). Group C contained seven ketones and five acids, which were mainly related to the genes in clusters I and VI. Among them, 2,3-pentanedione and 2-methyl-4-hexanone were superior, exhibiting strong correlations with 41 and 31 genes, respectively, including nine auxin response-related transcription factors or proteins, three bHLH transcription factors, three WRKY transcription factors and three β-ketoacyl-CoA synthases (Figure 6).

Alcohols and aldehydes (group D, 21 members) were closely related to the genes of clusters IV, V and VI. Among the alcohol volatiles, 2-butanol and 1-hexanol were highly correlated with 56 and 22 differentially expressed genes, respectively, including sixteen fatty acid synthesis genes, three ARF (*ARF3*, *ARF5,* and *ARF5-like*), three WRKY (*WRKY21*, *WRKY23,* and *WRKY6*), four bHLH (*bHLH143*, *bHLH48, bHLH68,* and *bHLH79*) and five MYB (M*YB3, MYB306, MYB86, MYB1R1,* and *EFM*) transcription factors (Figure 6). Aldehydes were the main components of group D, seven of which (propanal, methacrolein, 2-butenal, (Z)-2-heptenal, 5-ethylcyclopent-1-enecarboxaldehyde, ben-zaldehyde and heptadecanal) were strongly correlated with more than 20 DEGs, respectively, contributing 55.6% to the correlation in group D. After removing redundancy, seven aldehydes interacted with 95 differentially expressed genes, including 14 fatty acid metabolism related genes, eight bHLH transcription factors (*bHLH142, bHLH143, bHLH147, bHLH162, bHLH48, bHLH62, bHLH68,* and *bHLH79*), and 17 abscisic acid or auxin response factors [*ARF12, ARF3, ARF5-like, ARF6, ARF8, ARF8-like, ARF8-like2, IAA16, IAA27, IAA27-like, IAA4-like, SAUR52, GH3-1, PYL4* (abscisic acid receptor PYL4), *PYL8, PYL9,* and *ABF1*] (Figure 6). Consistent with group D, group E, consisted of four furans, four aromatic compounds and two miscellaneous volatiles, and also exhibited a strong correlation with the cluster VI genes. Fatty acid synthesis genes (*KCS1, KCS10, KCS11,* and *KCS11-like*), auxin response factors (*ARF12, ARF3, ARF6, ARF8, ARF8-like, IAA16, IAA27, IAA27-like, IAA29,* and *IAA4-like*) and bHLH transcription factors (*bHLH142, bHLH143, bHLH147, bHLH162, bHLH62,* and *bHLH79*) were the main genes interacting with the group E volatiles (Figure 6).

Esters (group F, 46 members) were mainly related to genes in cluster Ⅲ and cluster Ⅵ. In cluster III, fatty acid synthesis genes were dominant components, and were highly correlated with multiple esters. For example, *KCS1* gene was associated with 14 esters, such as cis-3-hexenyl hexanoate, hexyl acetate, isopentyl hexanoate and hexyl 2-methylbutyrate (Figure 6). Furthermore, 20 transcription factors in cluster VI, including four homeobox-leucine zipper proteins (*ATB13, ATHB16, ATHB16-like,* and *REV*), three bHLH transcription factors (*bHLH162, bHLH62,* and *bHLH79*) and one MYB transcription factor (*MYB50*), were identified to interact with esters such as isoamyl propionate, isobutyl isobutyrate and benzyl isobutyrate (Figure 6).

### 3.7 Verification of RNA sequencing results by qRT-PCR

To confirm the integrity of the RNA-Seq results, qRT-PCR experiments were performed on 9 selected genes (including six genes in the terpene or fatty acid metabolism pathways and three transcription factors) in the two varieties at 30 DAA and 50 DAA. The expression patterns of these genes were consistent with the RNA-seq results during fruit development (Supplementary Figure S5), indicating that the RNA data are valid and reliable.

## 4 Discussion

Aroma is an important quality characteristic of fruits and is mainly determined by the type and content of volatile substances in fruits (Dudareva et al., 2006). The volatile substances in pepper fruit are rich in variety, with esters, terpenes, aldehydes and alcohols as the dominant types (Kollmannsberger et al., 2011). In this study, the accumulation of esters, terpenes, alcohols and aldehydes showed obvious variety specificity. Esters and terpenoids including hexyl 2-methylbutyrate, 3-methylbutyric acid hexyl ester, 4-methylpentyl isovalerate, isopentyl isopentanoate, hexyl caprylate, (E)-β-famesene, β-elemen and α-cadinene were mainly accumulated in *C. chinense* QH. Furthermore, aldehydes and alcohols such as methacrolein, 2-propenal, isovaleraldehyde, isobutyraldehyde, 3-methyl-2-butenal, trans-2-pentenal, propanal, butanal, hexanal, 4-methyl-1-pentanol and 3-methyl-1-butanol were the dominant components of volatiles in WH fruit. Previous studies have shown that esters and terpenes are the main aroma contributors in *C. chinense* cultivars. Esters such as hexyl 2-methylbutyrate, 3-methylbutyric acid hexyl ester, 4-methylpentyl isovalerate and methyl salicylate have been identified as marker aroma compounds, leading to fruity aromas in the fruit of *C. chinense* cultivars (Heng et al., 2023; Huang et al., 2023). At present, a few aldehydes and alcohols have been identified as marker aroma compounds. (E)-2-hexenal and (Z)-3-hexenal have been reported to provide cherry tomatoes with strong green grass and green leaf odors (Selli et al., 2014). Moreover, the accumulation of aldehydes and alcohols induce the grassy aroma of immature peach fruit (Zhang et al., 2010; Niu et al., 2021). In summary, the large accumulation of esters and terpenoids with fruit aroma in QH fruit can explain the release of fruit aroma by QH fruit. However, although WH fruit exhibited high aldehyde and alcohol contents, the fruit did not emit green aroma. This may be because the content of these aldehydes and alcohols had not yet reached the corresponding aroma threshold. As the fruit developed, the type and content of volatiles in the QH and WH pepper varieties decreased to varying degrees. This agrees with Heng et al. (2023), who determined that the total volatile content decreased as the fruit changed from green to red. Therefore, compared with the physiological maturity stage, pepper fruit in the green maturity stage has a higher content of volatile substances such as esters, terpenes and aldehydes, and its fruit aroma and green grass odor are stronger. These properties may be more suitable for consumption.

The fatty acid metabolism pathway mainly includes four genes, namely, *LOX*, *HPL, ADH,* and *AAT*. The expressions of these genes play key roles in the synthesis of aldehydes, alcohols and esters (Beekwilder et al., 2004). *LOX* and *HPL* are two enzymes in the upstream of the fatty acid metabolism pathway, which can convert the precursor substances linoleic acid and linolenic acid into aldehydes through a two-step oxidation reaction (Huang et al., 2023). Recent studies have shown that four *LOX* genes (*MdLOX1*-*MdLOX4*) are positively correlated with the synthesis of aldehyde volatiles in apple (Liu et al., 2021). HPL is the last enzyme in the biosynthesis of aldehydes, and the loss of its function will directly affect the synthesis of aldehydes and alcohols. In *Nicotiana attenuate*, silencing the *NaHPL* gene reduced the release of (Z)-3-hexenal (Halitschke et al., 2004). The loss of the *HPL13* function led to a strong decrease in the total content of C6 volatiles in olive, accompanied by a decrease in the accumulation of hexanol, hexanal and (E)-2-hexanol (Cerezo et al., 2021). In this study, three LOX (*LOX1*, *LOX2* and *LOX4*) and two HPL (*HPL1* and *HPL2*) genes were highly expressed in the WH fruit at 30 DAA. This is consistent with the higher aldehyde content in WH fruit compared to QH fruit during fruit development, indicating that the aforementioned *LOX* and *HPL* genes play a key positive regulatory role in the biosynthesis of aldehydes. The aldehydes produced in the fatty acid and isoleucine metabolic pathways can be further converted into alcohols by alcohol dehydrogenase (ADH) (Wang et al., 2022b). Huang et al. (2023) detected 11 differentially expressed *ADH* genes in pepper, nine of which were transcribed at a higher level in *C. chinense* than in the *C. annuum* cultivated species. In this study, seven differentially expressed *ADH* genes were identified, but only *ADH2* and *ADH7* were highly expressed in the QH variety, while the transcription levels of the *ADH1*, *ADH3*, *ADH4,* and *ADH5* genes in QH were lower than those in WH. Moreover, we also observed that benzyl alcohol and 1-hexanol were highly accumulated in QH, while 3-methyl-1-butanol, (2E) -2-hexadecen-1-ol, 1-pentanol and 4-methyl-1-pentanol were highly accumulated in WH. This suggests that the ADH family members may have functional differentiation during evolution, and each family member may be responsible for the synthesis of different alcohols. Similar to the expression pattern of the *ADH* gene, four *AAT* genes were differentially expressed between the varieties, accompanied by the differential accumulation of esters. Terpene synthase determines the synthesis of different terpenoids in plants. In citrus, 95 genes were identified as potential *TPS* genes, of which the *STPS* genes were involved in the synthesis of monoterpenes, such as sabinene, D-limonene, linalool, trans-β-ocimene, β-myrcene and α-pinene (Zhang et al., 2020). The *MdAFS1* gene (terpene synthases) was reported to be responsible for the synthesis of sesquiterpene α-farnesene in apple (Liu et al., 2021). In this study, 13 differentially expressed *TPS* genes were identified. Among them, the expression pattern of seven *TPSs* (*TPS1*-*TPS6* and *TPS12*) was consistent with the accumulation pattern of sesquiterpenes, all of which had a higher transcriptional abundance or content in the QH variety. However, the remaining six *TPSs* (*TPS7*-*TPS11* and *TPS13*) were highly expressed in WH, accompanied by high monoterpene contents in this variety. The consistency of the *TPS* expression level and terpenoid content implies the critical involvement of *TPS1*-*TPS6* and *TPS12* in the synthesis of sesquiterpenes in pepper fruit, while the remaining seven *TPSs* genes (*TPS7*-*TPS11* and *TPS13*) may be primarily responsible for the synthesis of monoterpenes.

Previous studies have shown that transcription factors play a primary role in regulating volatile plant secondary metabolites (Lu et al., 2020). In petunia, three MYB transcription factors (ODO1, EOBII, and PhMYB4) regulate the accumulation of isoeugenol and eugenol by controlling the flux into alcohol/phenol metabolism pathways (Verdonk et al., 2005; Spitzer-Rimon et al., 2010; Colquhoun et al., 2011). The bHLH transcription factor MYC2-1 in apple was identified to regulate the synthesis of aldehydes in the early stage of fruit development and the synthesis of esters in the late stage of fruit development (Liu et al., 2021). MYB4 was predicted to be involved in the synthesis of cinnamyl acetate, benzyl acetate and eugenol in *Prunus mume* (Wang et al., 2022a). In patchouli (*Pogostemon cablin*), the transcription factors PcWRKY44 and PatSWC4 (MYB transcription factor) interacted with the jasmonic acid responsive protein JAZ4 to activate the expression of the patchoulol synthase gene (*PatPTS*) or farnesyl pyrophosphate synthase (*FPPS*), thus promoting the synthesis of sesquiterpene patchoulol (Chen et al., 2020; Wang et al., 2022b). However, the bHLH transcription factor PatGT-1 negatively regulates the synthesis of patchoulol by inhibiting the expression of *HMGR* genes in patchouli (Li et al., 2021b). Furthermore, MYC2 (bHLH) in *Arabidopsis thaliana* (Hong et al., 2012), MYB14 in conifer trees (Bedon et al., 2010), PbbHLH4 and PbbZIP4 in *Phalaenopsis bellina* (Ramya et al., 2020), and CgWRKY1 and CgWRKY2 in *Cymbidium goeringii* (Rchb.f.) Rchb.F.(Caputi and Aprea, 2011). have also been reported to be involved in the synthesis of terpenes. In this study, 96 transcription factors (including 12 bZIP, 11 bHLH, 11 MYB, 6 C3H, and 5 WRKY transcription factors) were significantly correlated with volatiles. Among them, transcription factors such as *bHLH62, bHLH143, bHLH147, MYB306, WRKY23,* and *bZIP9* were significantly correlated with multiple volatiles and were highly expressed in the QH or WH varieties. This indicates that these transcription factors may be involved in the synthesis of aroma components by regulating the expression of key genes in the volatile synthesis pathway of pepper fruit.

Previous research has demonstrated the involvement of plant hormones in regulating the synthesis of plant volatiles (Wu et al., 2018). Exogenous auxin and abscisic acid can change the volatile profile of tomato fruit by affecting the expression of volatile metabolic pathway genes (Wu et al., 2018; Tobaruela et al., 2021). In patchouli (*Pogostemon cablin*), the ethylene response factor PatDREB interacted with the MYB transcription factor PatSWC4 to activate the expression of the patchoulol synthase gene *PatPTS*, thus promoting the synthesis of sesquiterpene patchoulol (Chen et al., 2022). The ethylene response factor CitERF71 regulates the synthesis of E-geraniol (E) by activating the expression of the *CitTPS16* gene in citrus fruit (Li et al., 2017). The jasmonic acid response factor ORCA3 has been linked to the production of terpenoid indole alkaloids in *Catharanthus roseus* (van der Fits and Memelink, 2000). The auxin-responsive protein HcIAA4 activates the expression of *HcTPS5* by interacting with HcMYB1, thereby regulating the synthesis of linalool and methyl benzoate in *Hedychium coronarium* (Ke et al., 2021). In this study, 37 hormone response factors or hormone synthetic structure genes were identified to be significantly correlated with volatiles, including 21 auxin response factors. Among them, auxin response factors such as *IAA27, IAA27-like, ARF8, ARF8-like, ARF12,* and *ARF3* were closely linked to more than 10 volatiles, respectively, and were located at key nodes in the interaction network of differentially expressed genes and metabolites. This suggests that these auxin response factors play a crucial role in regulating the synthesis of aroma substances. At present, the mechanism of auxin regulating volatile synthesis remains unclear. However, in this study, a large number of auxin response factors were enriched in the interaction network, providing new tools to analyze the mechanism underlying the auxin regulation of volatile synthesis. The specific functions of these auxin factors in volatile synthesis will be the focus of future studies.

## 5 Conclusion

Esters, aldehydes, alcohols and terpenes were the dominant volatile components in pepper fruits, and their contents decreased with the ripening of pepper fruits. The higher accumulation of esters and terpenes may be responsible for the release of fruit aroma by QH fruit. The biosynthesis of volatile compounds was closely related to fatty acid and terpenoid metabolic pathways, and the high expression of genes in fatty acid and terpenoid metabolic pathways was key in determining the accumulation of volatiles in pepper fruit. Furthermore, we constructed a regulatory network of volatile biosynthesis, identified potential candidate genes that may be involved in the biosynthesis of volatile compounds, and found that auxin may regulate the accumulation of volatiles in pepper fruits by inducing the high expression of auxin-responsive proteins or factors. These results have established a practical basis for further exploration of the regulatory mechanisms underlying the formation of aroma compounds in pepper.

## Data Availability

The original contributions presented in the study are publicly available. This data can be found here: https://www.ncbi.nlm.nih.gov/search/all/?term=PRJNA1014701.
