# Supplementary figures and images for: Integrative analysis of non-targeted metabolome and transcriptome reveals the mechanism of volatile formation in pepper fruit
Source: Front Genet. 2023 Nov 10;14:1290492. doi: 10.3389/fgene.2023.1290492 (PMC10667453; doi:10.3389/fgene.2023.1290492)

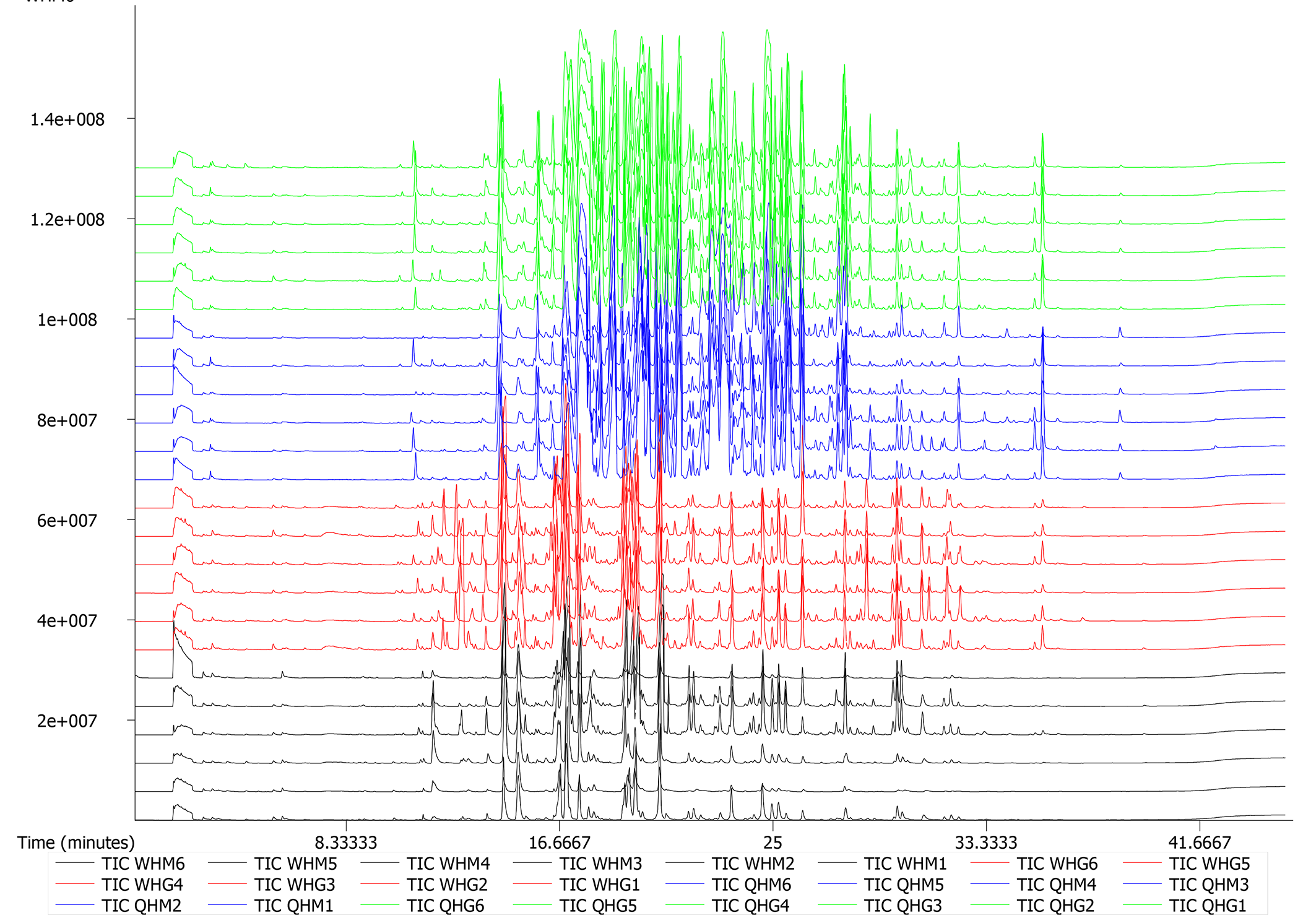

Supplement: Supplementary file 2 [file DataSheet2.ZIP › Supplementary Figure/Figure S1.tif]

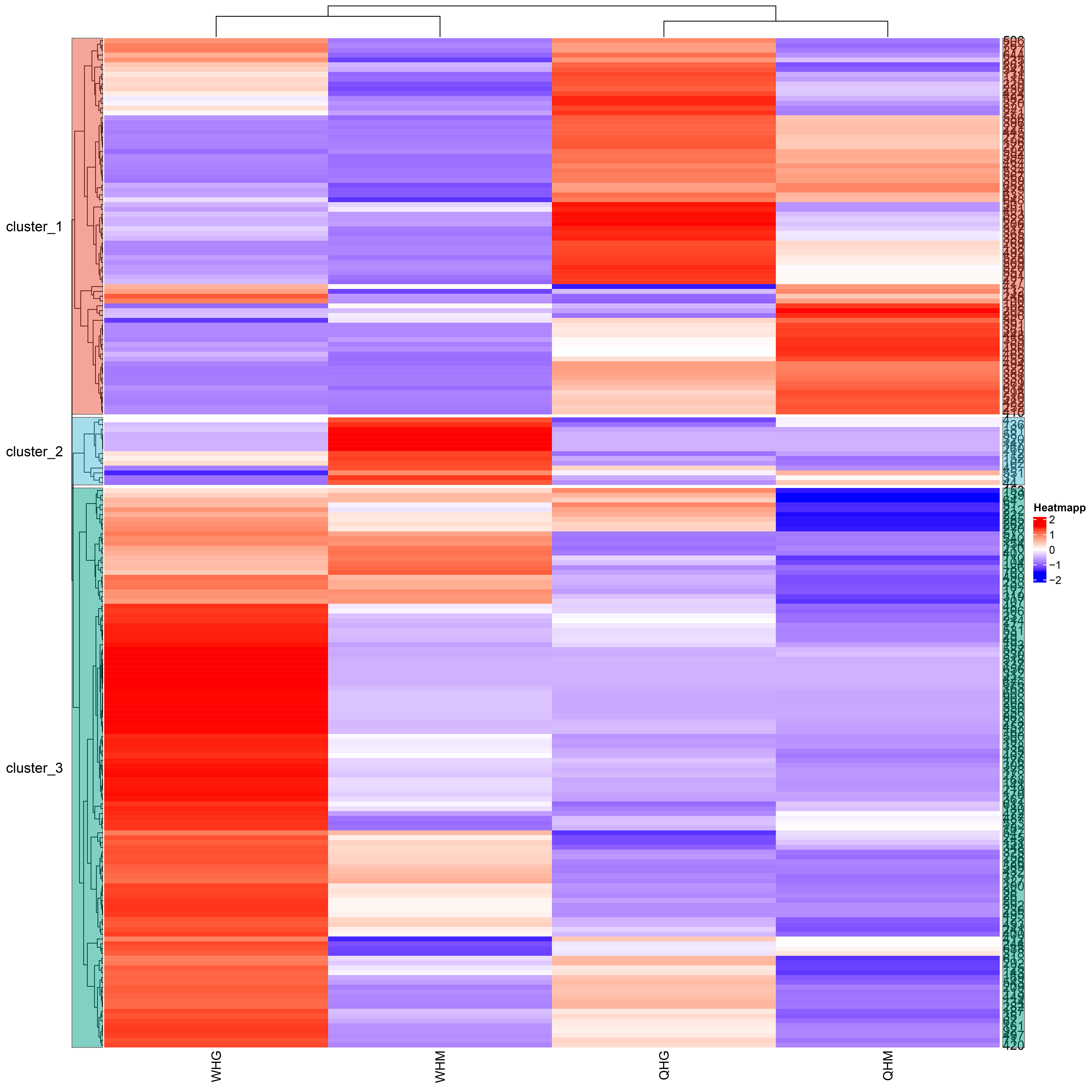

Supplement: Supplementary file 2 [file DataSheet2.ZIP › Supplementary Figure/Figure S2.tif]

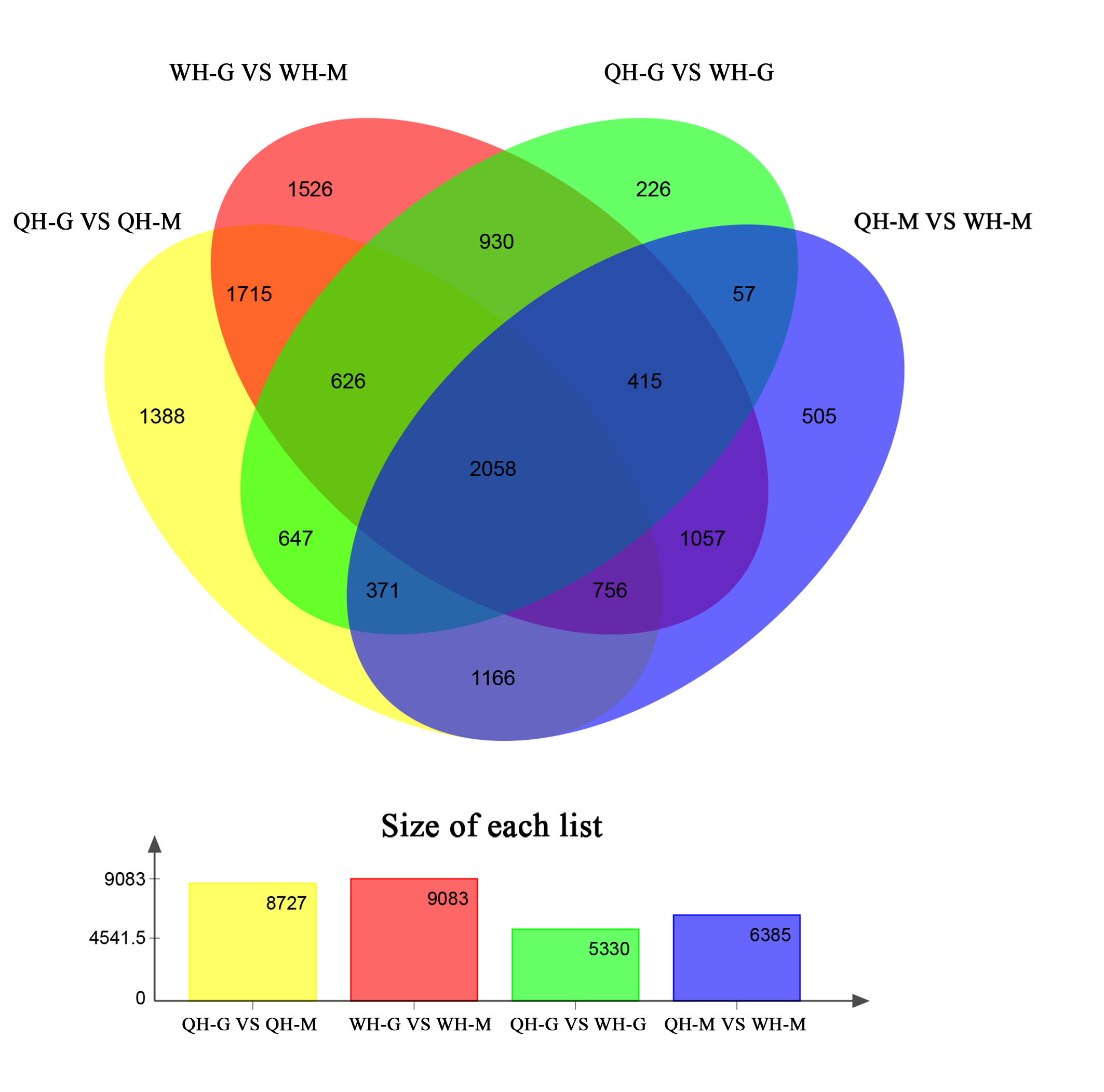

Supplement: Supplementary file 2 [file DataSheet2.ZIP › Supplementary Figure/Figure S3.tif]

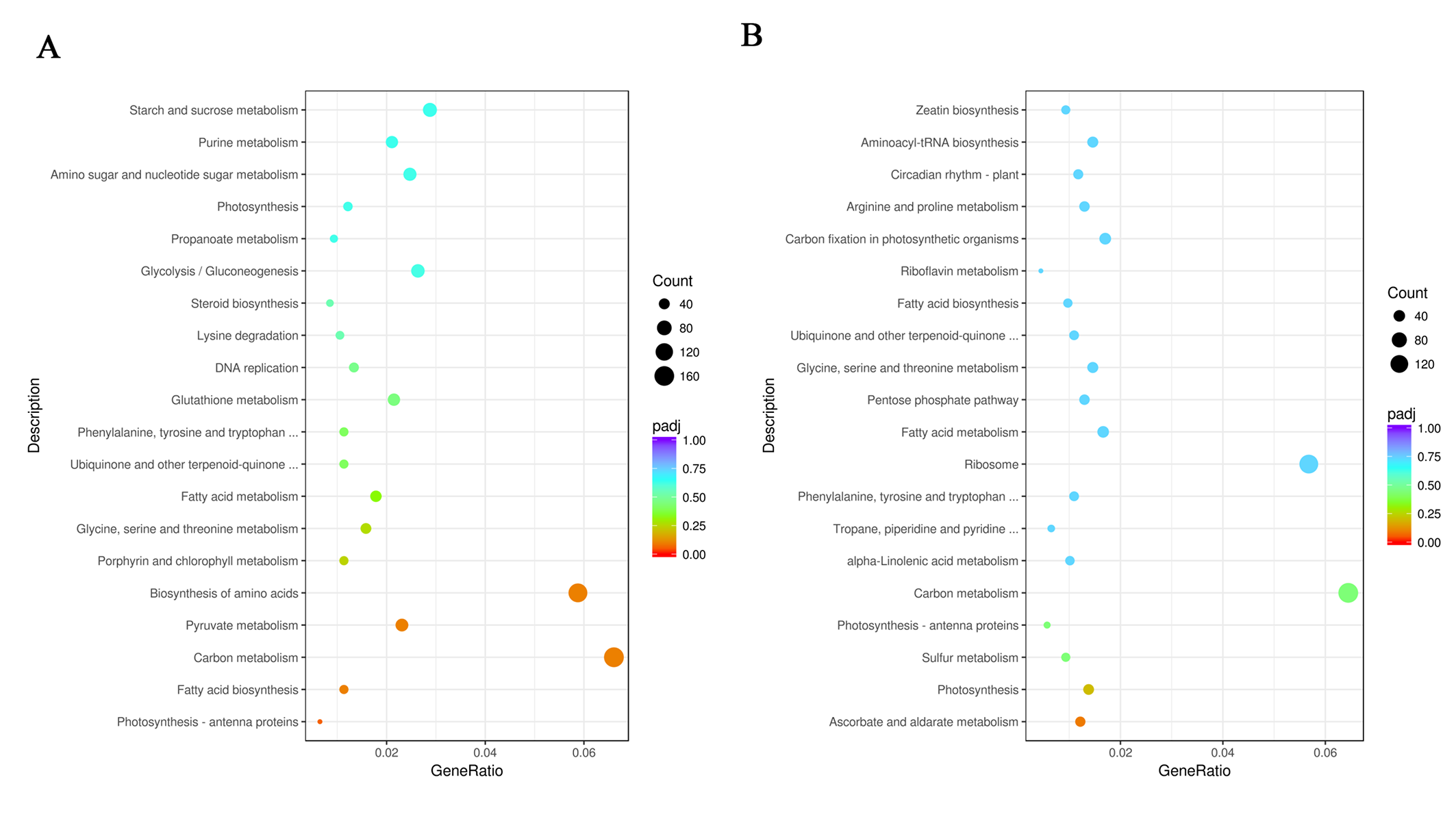

Supplement: Supplementary file 2 [file DataSheet2.ZIP › Supplementary Figure/Figure S4.tif]

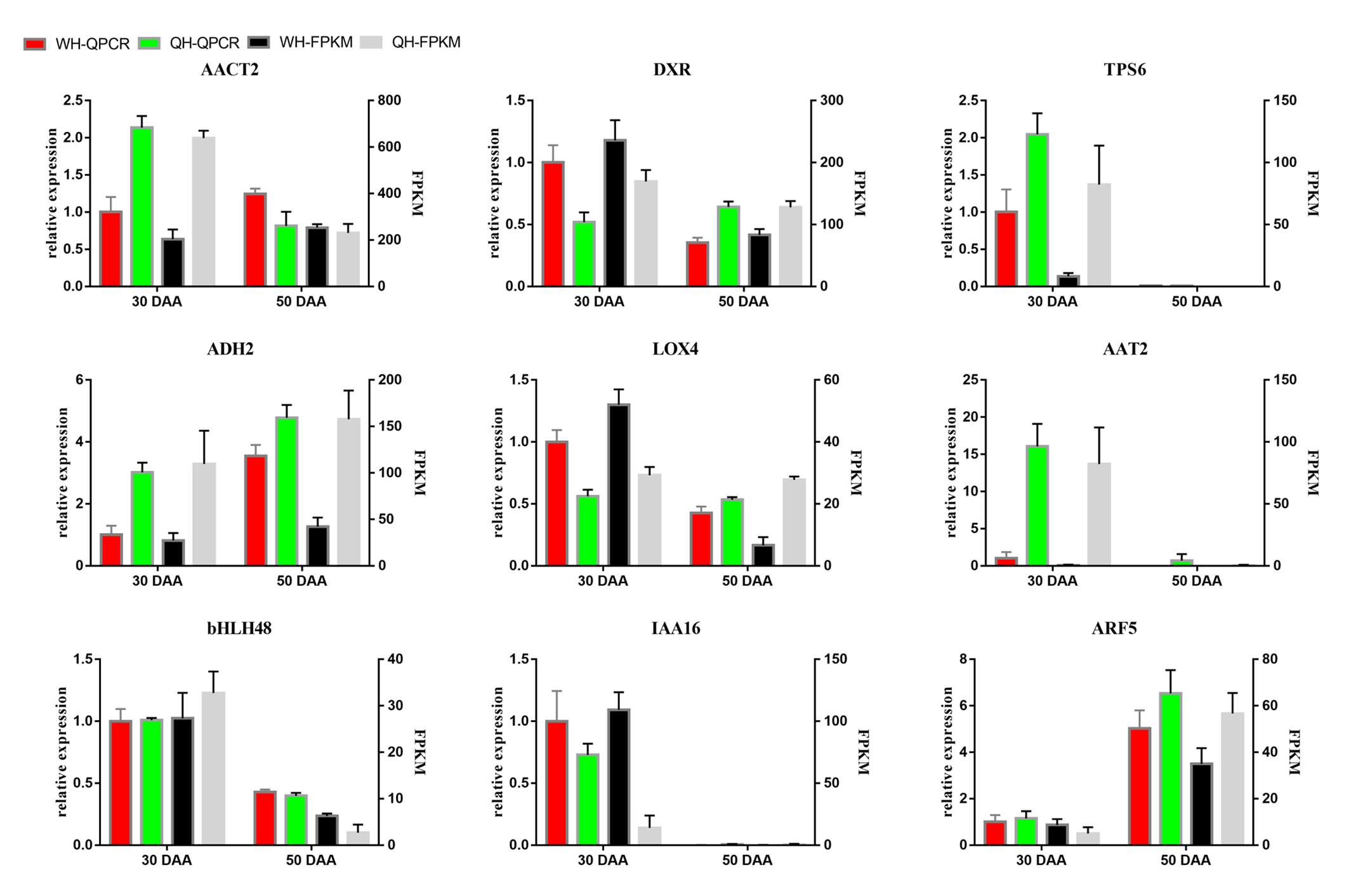

Supplement: Supplementary file 2 [file DataSheet2.ZIP › Supplementary Figure/Figure S5.tif]
